# Akt1 in Osteoblasts and Osteoclasts Controls Bone Remodeling

**DOI:** 10.1371/journal.pone.0001058

**Published:** 2007-10-24

**Authors:** Naohiro Kawamura, Fumitaka Kugimiya, Yasushi Oshima, Shinsuke Ohba, Toshiyuki Ikeda, Taku Saito, Yusuke Shinoda, Yosuke Kawasaki, Naoshi Ogata, Kazuto Hoshi, Toru Akiyama, William S. Chen, Nissim Hay, Kazuyuki Tobe, Takashi Kadowaki, Yoshiaki Azuma, Sakae Tanaka, Kozo Nakamura, Ung-il Chung, Hiroshi Kawaguchi

**Affiliations:** 1 Department of Sensory and Motor System Medicine, Faculty of Medicine, University of Tokyo, Tokyo, Japan; 2 Center for Disease Biology and Integrative Medicine, Faculty of Medicine, University of Tokyo, Tokyo, Japan; 3 Transgenic and Knockout Mouse Laboratory, University of Kansas, Lawrence, Lawrence, Kansas, United States of America; 4 Department of Molecular Genetics, College of Medicine, University of Illinois at Chicago, Chicago, Illinois, United States of America; 5 Department of Metabolic Diseases, Faculty of Medicine, University of Tokyo, Tokyo, Japan; 6 Teijin Institute for Biomedical Research, Tokyo, Japan; Baylor College of Medicine, United States of America

## Abstract

Bone mass and turnover are maintained by the coordinated balance between bone formation by osteoblasts and bone resorption by osteoclasts, under regulation of many systemic and local factors. Phosphoinositide-dependent serine-threonine protein kinase Akt is one of the key players in the signaling of potent bone anabolic factors. This study initially showed that the disruption of Akt1, a major Akt in osteoblasts and osteoclasts, in mice led to low-turnover osteopenia through dysfunctions of both cells. Ex vivo cell culture analyses revealed that the osteoblast dysfunction was traced to the increased susceptibility to the mitochondria-dependent apoptosis and the decreased transcriptional activity of runt-related transcription factor 2 (Runx2), a master regulator of osteoblast differentiation. Notably, our findings revealed a novel role of Akt1/forkhead box class O (FoxO) 3a/Bim axis in the apoptosis of osteoblasts: Akt1 phosphorylates the transcription factor FoxO3a to prevent its nuclear localization, leading to impaired transactivation of its target gene Bim which was also shown to be a potent proapoptotic molecule in osteoblasts. The osteoclast dysfunction was attributed to the cell autonomous defects of differentiation and survival in osteoclasts and the decreased expression of receptor activator of nuclear factor-κB ligand (RANKL), a major determinant of osteoclastogenesis, in osteoblasts. Akt1 was established as a crucial regulator of osteoblasts and osteoclasts by promoting their differentiation and survival to maintain bone mass and turnover. The molecular network found in this study will provide a basis for rational therapeutic targets for bone disorders.

## Introduction

Bone is continually remodeled according to physiological circumstances through bone formation by osteoblasts and bone resorption by osteoclasts, and bone mass and turnover are maintained by their coordinated balance in healthy adults. Many systemic and local factors are involved in the regulation [1], [2], among which insulin, insulin-like growth factor-I (IGF-I), bone morphogenetic factors (BMPs), and Wnt proteins are potent bone anabolic factors [3]–[6]. A serine-threonine kinase Akt, also called protein kinase B (PKB), is known as a potent signal transducer of these bone anabolic factors [7]–[9].

There are three Akt family members, Akt1/PKBα, Akt2/PKBβ and Akt3/PKBγ. Akt1 and Akt2, but not Akt3, are reported to be ubiquitously and similarly expressed in various tissues, although Akt2 is expressed more predominantly in insulin target tissues such as fat, liver and muscle [9], [10]. Accordingly, Akt1-/- mice and Akt2-/- mice show similar phenotypes including dwarfism, except that only the latter exhibit severe diabetes [10]–[13]. Although phenotype of mice lacking single Akt isoform is relatively mild possibly due to functional redundancy of the remaining isoforms, double-knockout of Akt1 and Akt2 in mice causes severely impaired bone development and death shortly after birth [14]. The abnormalities of these knockout mice certainly imply a crucial commitment of Akt signals to bone metabolism, yet it has remained unknown whether or not these were due to the cell autonomous effects of the Akt deficiency on bone cells. To elucidate the physiological role of the Akt signaling in the regulation of bone formation and resorption, the present study initially analyzed the bones of mice lacking Akt1, a major Akt isoform in bone, and found that the deficiency caused osteopenia with a low turnover state. We further investigated the underlying cellular and molecular mechanisms in osteoblasts and osteoclasts.

## Results

### Akt1 is a major isoform in bone cells

We initially confirmed that the total Akt (Akt1–3) was phosphorylated by osteogenic factors IGF-I and BMP-2 in cultured osteoblasts, and by a bone resorptive macrophage colony-stimulating factor (M-CSF) in osteoclast-progenitor bone marrow macrophages (BMM) and mature osteoclasts (Supp. Fig S1A). Among the three Akt isoforms, Akt1 and Akt2 were highly expressed in these bone cells (Supp. Fig S1B).

### Akt1 deficiency causes decreased bone mass and formation

**Figure 1:**
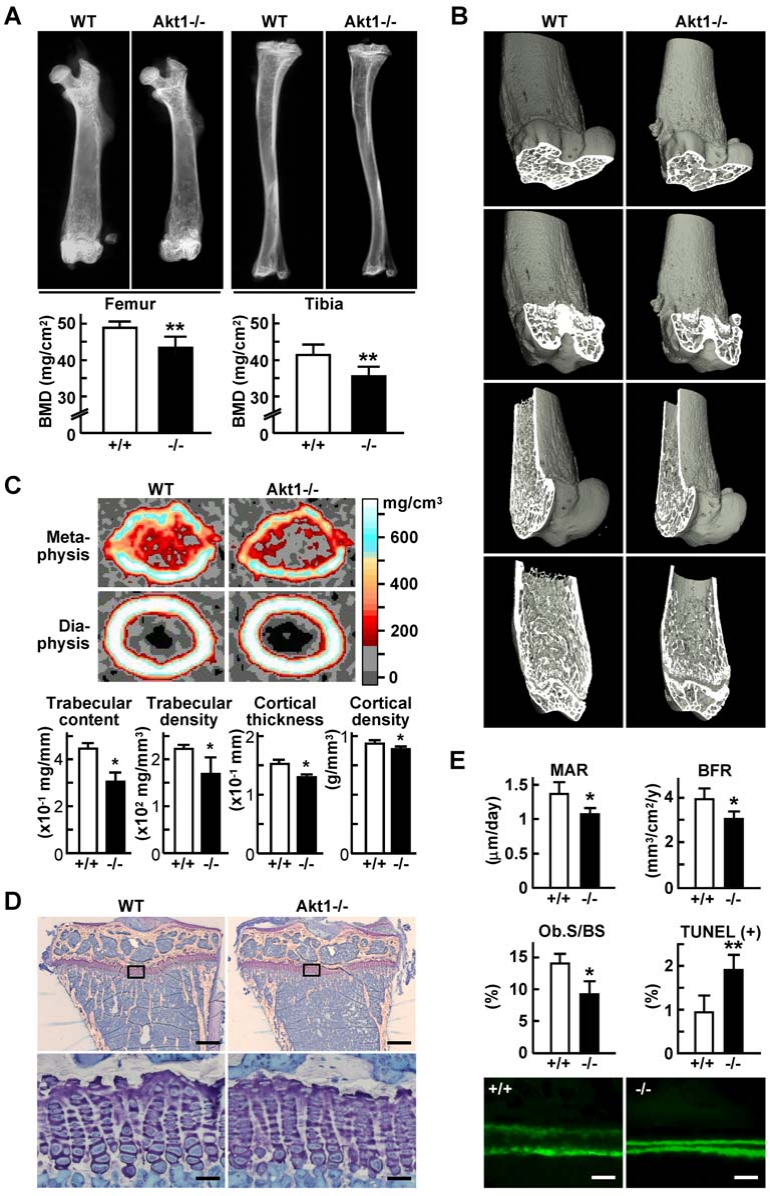
Bone mass and formation are decreased in Akt1-/- mice. (A) Plain X-ray images of femur and tibia in WT and Akt1-/- male littermates at 8 weeks of age. The BMD of the entire femurs and tibias measured by DXA is shown in the graphs below. Data are expressed as means (bars)±SEM (error bars) for 5 WT and 3 Akt1-/- littermates. **P<0.01 vs. WT. (B) Three-dimensional CT images of distal femurs. (C) pQCT images of the distal metaphysis and the mid-diaphysis of the femurs. The color gradient indicating BMD is shown in the right bar. The trabecular content and density at the metaphysis, and the cortical thickness and density at the diaphysis are shown in the graphs below. Data are expressed as means (bars)±SEM (error bars) for 4 mice/group. *P<0.05 vs. WT. (D) Toluidine blue staining of the proximal tibias. Inset boxes indicate the regions of the bottom figures. The growth plate heights were 94.7±2.9 and 95.8±3.8 µm for WT and Akt1-/-, respectively (mean±SEM of 4 mice/group). Bars, 200 µm (top)&30 µm (bottom). (E) Bone formation parameters in histomorphometric analysis at the proximal tibias. MAR, mineral apposition rate; BFR/BS, bone formation rate per bone surface; Ob.S/BS, osteoblast surface per bone surface; TUNEL(+), percentage of TUNEL-positive apoptotic osteoblasts. Data are expressed as means (bars)±SEM (error bars) for 3–4 mice/group. *P<0.05, **P<0.01 vs. WT. The bottom figures show the representative calcein double labelings; bars, 20 µm.

To investigate the physiological role of the Akt signaling in bone, we examined the bone phenotype of Akt1 deficient (Akt1-/-) mice, because Akt1; Akt2 double-knockout mice die shortly after birth [14]. Akt1-/- mice were healthy and fertile without abnormality in major organs or skeletal patterning, but were smaller in size compared to the wild-type (WT) littermates (Supp. Fig S2) as previously reported [11]. Radiological analyses of long bones in 8 week-old mice using plain X-ray, bone mineral density (BMD) analysis (Fig 1A), three dimensional (3D) CT (Fig 1B), and peripheral quantitative CT (pQCT) (Fig 1C) revealed decreases in both trabecular and cortical bones as a result of the Akt1 deficiency. Although the growth plate was not affected (Fig 1D), bone formation parameters (MAR&BFR) of the trabecular bone were decreased (Fig 1E), indicating that osteopenia in the Akt1-/- mice was at least partly due to the impairment of osteoblastic bone formation. This defect was accompanied by a decrease in the number of osteoblasts shown by the percentage of bone surface covered by cuboidal osteoblasts (Ob.S/BS) and an increase in that of apoptotic osteoblasts shown by terminal deoxynucleotidyl transferase mediated dUTP nick-end-labeling (TUNEL)-positive cells (Fig 1E).

### Akt1 deficiency enhances susceptibility to apoptosis of osteoblasts via FoxO3a

**Figure 2:**
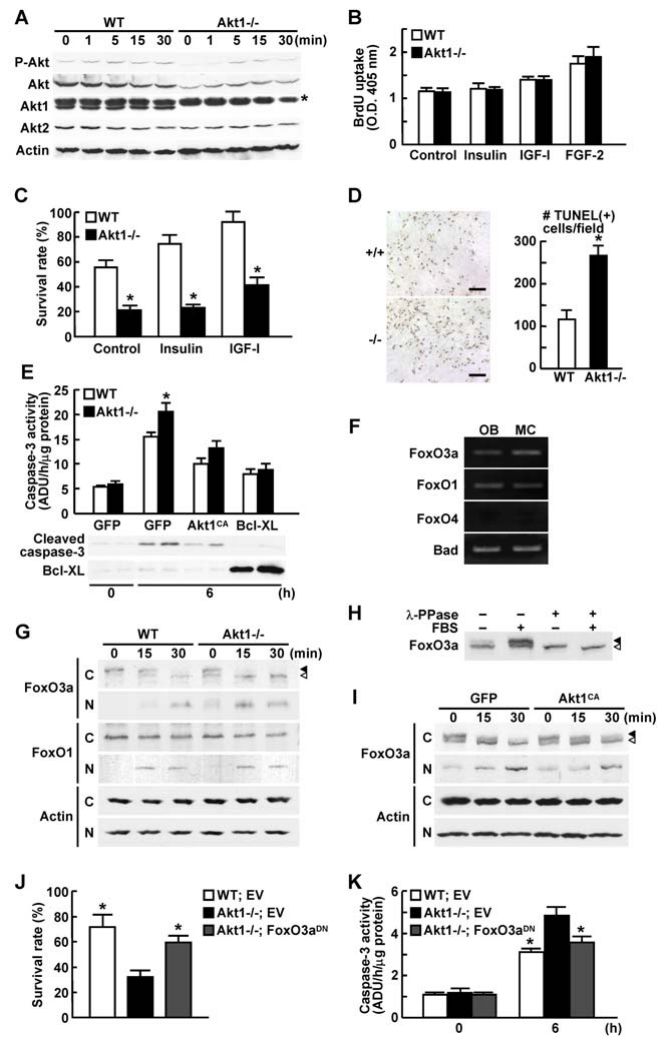
Akt1-/- osteoblasts are susceptibile to apoptosis via FoxO3a. (A) Time course of phosphorylated total Akt (P-Akt), total Akt (Akt), Akt1, Akt2, and β-actin levels after IGF-I stimulation determined by Western blotting in cultured calvarial osteoblasts from WT and Akt1-/- mice. An asterisk indicates a nonspecific band detected by an antibody to Akt1. (B) Cell proliferation determined by BrdU uptake into calvarial osteoblasts of two genotypes cultured with and without insulin, IGF-I, or FGF-2. Data are expressed as means (bars)±SEM (error bars) for 6 wells/group. (C) Survival rate of WT and Akt1-/- osteoblasts 48 h after serum deprivation in cultures with and without insulin or IGF-I. Data are expressed as means (bars)±SEM (error bars) for 6 wells/group. *P<0.01 vs. WT. (D) The number of TUNEL-positive osteoblasts per field (6×10^5^ µm^2^) 24 h after serum deprivation in cultured calvarial osteoblasts. Data are expressed as means (bars)±SEM (error bars) of 3 wells/group. *P<0.01 vs. WT. Bars, 100 µm. (E) Caspase-3 activity (top graph) and Western blotting for cleaved caspase-3 and Bcl-x_L_ (bottom) in WT and Akt1-/- osteoblasts adenovirally transfected with GFP, Akt1^CA^, and Bcl-x_L_ before and 6 h after serum deprivation. Data are expressed as means (bars)±SEM (error bars) for 3 wells/group. *P<0.05 vs. WT. ADU: arbitrary densitometry unit. (F) FoxO3a, FoxO1, FoxO4 and Bad expressions determined by RT-PCR in primary mouse calvarial osteoblasts (OB) and MC3T3-E1 cells (MC). (G) Time course of FoxO3a, FoxO1, and β-actin levels by Western blotting after serum deprivation in the cytoplasmic (C) and nuclear (N) fractions of cultured WT and Akt1-/- osteoblasts. Closed and open arrowheads indicate phosphorylated and unphosphorylated FoxO3a, respectively. (H) Western blotting for FoxO3a in the cytoplasmic fraction of WT osteoblasts cultured with and without 10% FBS for 30 min. The lysates were treated with and without lambda protein phosphatase (λ-PPase). (I) Time course of FoxO3a and β-actin levels by Western blotting after serum deprivation in the cytoplasmic (C) and nuclear (N) fractions of cultured osteoblasts adenovirally transfected with GPF or Akt1^CA^. (J) Survival rate of WT and Akt1-/- osteoblasts retrovirally transfected with empty vector (EV) or FoxO3a^DN^ 48 h after serum deprivation. Data are expressed as means (bars)±SEM (error bars) for 6 wells/group. *P<0.05 vs. Akt1-/- with EV. (K) Caspase-3 activity of WT and Akt1-/- osteoblasts retrovirally transfected with EV or FoxO3a^DN^ before and 6 h after serum deprivation. Data are expressed as means (bars)±SEM (error bars) for 3 wells/group. *P<0.05 vs. Akt1-/- with EV. ADU: arbitrary densitometry unit.

To learn the mechanism of impaired bone formation, we compared ex vivo functions of osteoblasts isolated from WT and Akt1-/- littermates. We first confirmed that both phosphorylated and unphosphorylated Akt proteins were considerably decreased in cultured Akt1-/- osteoblasts without compensatory increase of Akt2 protein, indicating a substantial suppression of the total Akt signaling by depletion of an isoform Akt1 in osteoblasts (Fig 2A). Although cell proliferation was not affected (Fig 2B), cell survival after serum deprivation was decreased by the Akt1 deficiency (Fig 2C). This decrease was confirmed to be due to the enhanced susceptibility to apoptosis of osteoblasts by TUNEL staining (Fig 2D). Caspase-3 activity and the cleaved active form was enhanced in Akt1-/- osteoblasts, which was restored by adenoviral introduction of a constitutively active form of Akt1 (Akt1^CA^) or Bcl-x_L_, a specific inhibitor of mitochondria-dependent apoptosis [15] (Fig 2E). Hence, Akt1 deficiency in osteoblasts is not likely to affect the proliferation, but suppresses the survival through an enhanced susceptibility to mitochondria-dependent apoptosis.

Among apoptosis-related molecules that have been reported to be possible substrates of Akt [9], [16], transcription factors FoxO3a and FoxO1, and a proapoptotic molecule Bad were expressed in primary osteoblasts and osteoblastic cell line MC3T3-E1 (Fig 2F). Since phosphorylation of FoxO proteins is known to result in their nuclear exclusion in other types of cells [16], we confirmed the nuclear entry of FoxO3a after serum deprivation in MC3T3-E1 cells (Supp. Fig S3). The nuclear localization of FoxO3a was more enhanced in Akt1-/- osteoblasts than in WT after serum deprivation (Fig 2G). This correlated with the decrease of the upper band and the increase of the lower band in the cytoplasmic fraction of the immunoblotting, which were proved to be phosphorylated and unphosphorylated FoxO3a, respectively, since the upper one was enhanced by FBS and eliminated by lambda protein phosphatase (Fig 2H). Meanwhile, Akt1 overexpression by Akt1^CA^ transfection in osteoblasts stimulated the cytoplasmic FoxO3a phosphorylation and inhibited the nuclear entry (Fig 2I). Contrarily, subcellular localization of FoxO1 was not affected by the Akt1 deficiency (Fig 2G), nor was phosphorylated Bad protein detected in osteoblasts, probably due to its low level (data not shown). Finally, the decreased survival rate and the increased caspase-3 activity of Akt1-/- osteoblasts after serum deprivation were restored by introduction of a dominant-negative form of FoxO3a (FoxO3a^DN^) (Fig 2J, K).

The lines of results indicate that a transcription factor FixO3a is a phosphorylation target of Akt1 in the anti-apoptotic action on osteoblasts, so that the Akt1 deficiency attenuates FoxO3a phosphorylation and nuclear exclusion, which leads to the enhanced transcriptional activity somehow causing apoptosis.

### Akt1 suppresses osteoblast apoptosis through inhibition of FoxO3a and Bim

**Figure 3:**
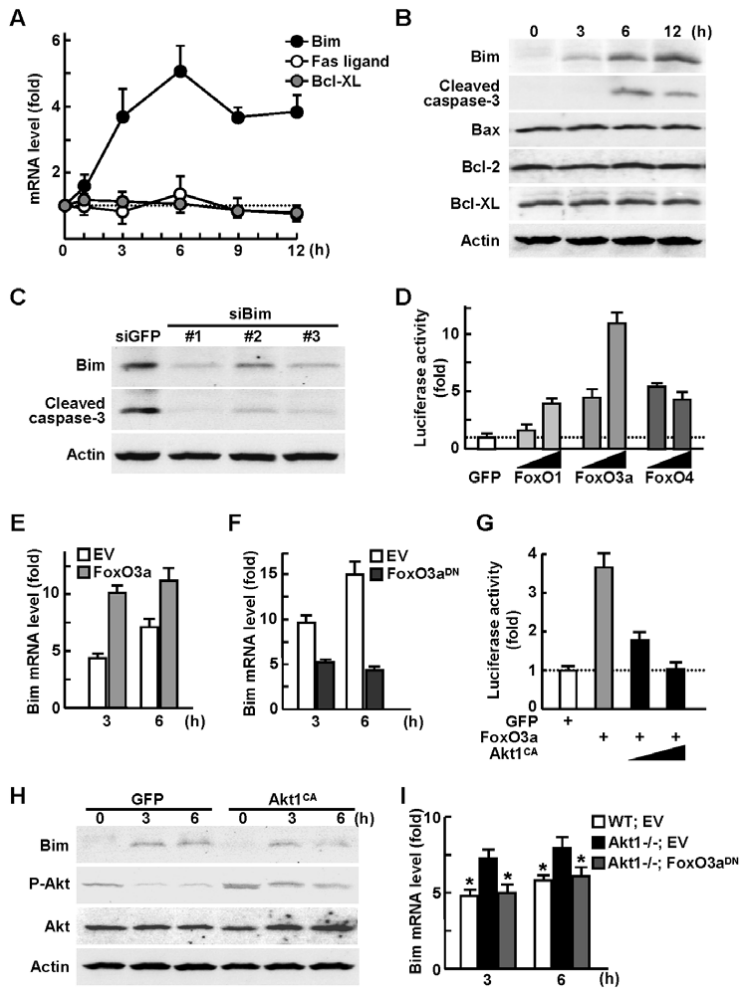
Akt1 suppresses osteoblast apoptosis via inhibition of FoxO3a and Bim. (A) Time course of mRNA levels determined by real-time RT-PCR of Bim, Fas ligand, and Bcl-x_L_ after serum deprivation in cultured calvarial osteoblasts. Data are normalized to those of β-actin and are expressed as means (symbols)±SEM (error bars) of the relative amount compared to time 0. (B) Time course of Bim, cleaved caspase-3, Bax, Bcl-2, Bcl-x_L_, and β-actin levels determined by Western blotting after serum deprivation in cultured calvarial osteoblasts. (C) Western blotting for Bim, cleaved caspase-3, and β-actin levels 6 h after serum deprivation in MC3T3-E1 cells retrovirally transfected with three kinds of Bim siRNA or the control GFP siRNA. (D) Bim promoter activity determined by luciferase reporter assay in cultured MC3T3-E1 cells transfected with luciferase reporter constructs containing a 2-Kb Bim 5′-end flanking region. Plasmid vectors of the control GFP, FoxO1, FoxO3a, and FoxO4 were co-transfected in increasing amounts. Data are expressed as means (bars)±SEM (error bars) of the fold change compared to GFP. (E, F) Bim mRNA level determined by real-time RT-PCR 3 h and 6 h after serum deprivation in MC3T3-E1 cells retrovirally transfected with FoxO3a (E), FoxO3a^DN ^(F), or the respective EV. Data are normalized to those of β-actin and are expressed as means (bars)±SEM (error bars) of the relative amount compared to time 0. (G) Bim promoter activity analysis in MC3T3-E1 cells transfected with the control GFP, FoxO3a, and Akt1^CA^ plasmid vectors in increasing amounts. Data are expressed as means (bars)±SEM (error bars) of the fold change compared to GFP. (H) Time course of Bim, phosphorylated total Akt (P-Akt), total Akt (Akt), and β-actin levels determined by Western blotting after serum deprivation in cultured calvarial osteoblasts adenovirally transfected with GPF or Akt1^CA^. (I) Bim mRNA level determined by real-time RT-PCR 3 h and 6 h after serum deprivation in cultured WT and Akt1-/- osteoblasts retrovirally transfected with EV or FoxO3a^DN^. Data are normalized to those of β-actin and are expressed as means (bars)±SEM (error bars) of the relative amount compared to time 0. *P<0.05 vs. Akt1-/- with EV.

We next sought to identify the transcriptional target of FoxO3a that lies downstream of the Akt1 anti-apoptotic signaling in osteoblasts. Among the candidate molecules Fas ligand, Bim, and Bcl-x_L_ that were reported previously in other tissues [17], only Bim mRNA level was increased after serum deprivation in osteoblasts (Fig 3A). Bim protein level was also increased, followed by the induction of cleaved caspase-3, while none of the major regulators of mitochondria-dependent apoptosis: proapoptotic Bax, anti-apoptotic Bcl-2 and Bcl-x_L_ [15], was affected by serum deprivation (Fig 3B). The cleaved caspase-3 induction was mediated by Bim, since it was suppressed by silencing of Bim through RNA interference (Fig 3C). Actinomycin D, an inhibitor of RNA transcripion, canceled the Bim increase after serum deprivation, indicating that this is a transcriptional induction (Supp. Fig S4A, B). The Bim promoter activity was actually enhanced by FoxOs, among which FoxO3a exhibited the strongest transactivity (Fig 3D). Endogenous Bim expression was confirmed to be enhanced by FoxO3a overexpression and attenuated by the suppression (Fig 3E, F). These results indicate that Bim is a transcriptional target of FoxO3a in osteoblasts.

We then looked at the involvement of Akt1 in the FoxO3a and Bim interaction in osteoblasts. Gain-of-function of Akt1 by Akt1^CA^ transfection abrogated the stimulation of the Bim promoter activity by FoxO3a (Fig 3G). The induction of endogenous Bim after serum deprivation was also suppressed by Akt1^CA^ (Fig 3H) as well as by insulin or IGF-I, potent activators of the Akt signaling (Supp. Fig S4C). Finally, the Bim expression in Akt1-/- osteoblasts was higher than that in WT osteoblasts, which was restored by the FoxO3a^DN^ transfection (Fig 3I).

Taken together, we conclude that Akt1 phosphorylates FoxO3a to prevent it from translocating into nucleus, which leads to suppression of Bim transactivation and osteoblast apoptosis.

### Akt1 deficiency impairs Runx2-dependent differentiation and function of osteoblasts

**Figure 4:**
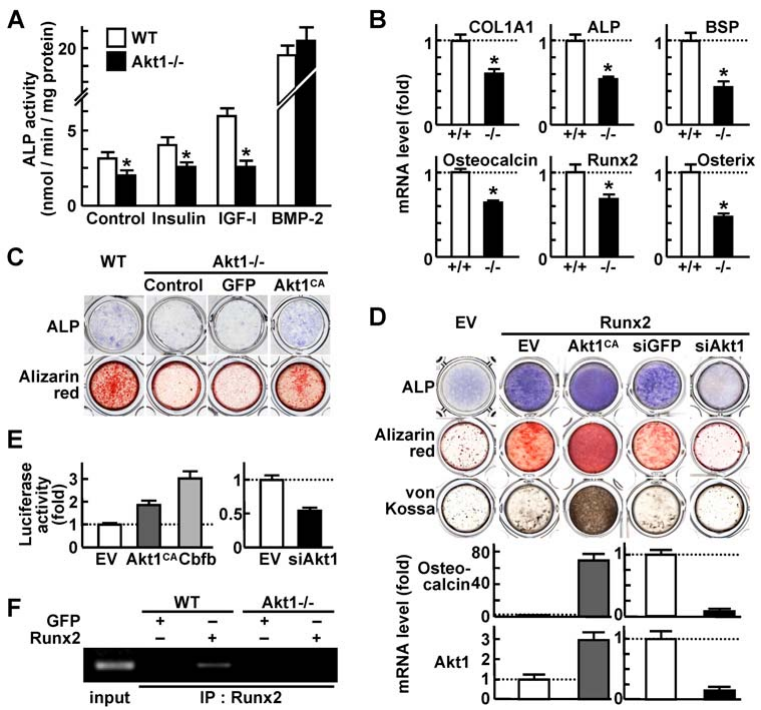
Akt1 enhances Runx2-dependent osteoblast differentiation and function. (A) ALP activity of WT and Akt1-/- osteoblasts cultured with and without insulin, IGF-I or BMP-2. Data are expressed as means (bars)±SEM (error bars) for 4 wells/group. *P<0.05 vs. WT. (B) mRNA levels of type I collagen (COL1A1), ALP, bone sialoprotein (BSP), osteocalcin, Runx2, and osterix in WT and Akt1-/- osteoblasts determined by real-time RT-PCR. Data are normalized to those of β-actin and are expressed as means (bars)±SEM (error bars) of the relative amount compared to WT culture. *P<0.01 vs. WT. (C) ALP and Alizarin red stainings of WT and Akt1-/- osteoblasts with and without adenoviral transfection of GFP or Akt1^CA^. (D) ALP, Alizarin red, and von Kossa stainings of MC3T3-E1 cells retrovirally transfected with Runx2 or the empty vector (EV). Runx2 transfectants were retrovirally co-transfected with Akt1^CA^ or the control EV, and Akt1 siRNA (siAkt1) or the control (siGFP). The graphs indicate mRNA levels of osteocalcin and Akt1 determined by real-time RT-PCR. Data are normalized to those of β-actin and are expressed as means (bars)±SEM (error bars) of the relative amount compared to the control culture. (E) Osteocalcin promoter activity in retroviral Runx2 transfectants of MC3T3-E1 cells that were transfected with luciferase reporter constructs containing a 1,050 bp osteocalcin 5′-end flanking region. Plasmid vectors of Akt1^CA^, the negative control EV, and the positive control Cbfb that is a representative co-activator of Runx2, as well as those of siAkt1 and the control EV were co-transfected. Data are expressed as means (bars)±SEM (error bars) of the fold change compared to EV. (F) ChIP assay using cell lysates of WT and Akt1-/- osteoblasts adenovirally transfected with GFP or Runx2. Purified DNA from with (IP) and without (input) immunoprecipitation by an antibody to Runx2 was amplified by PCR using a primer set in the mouse osteocalcin promoter region (−471/−67).

Since the in vivo histomorphometric analysis of the Alt1-/- bone (Fig 1E) showed not only a decrease in osteoblast number, but also a decrease in the ability of an individual osteoblast to form bone (MAR), we performed ex vivo analyses of the effects of Akt1 deficiency on osteoblast differentiation and function, in addition to their proliferation and apoptosis. The differentiation determined by alkaline phosphatase (ALP) activity was impaired in Akt1-/- osteoblasts in the control and stimulated cultures by insulin or IGF-I, but not by BMP-2 (Fig 4A). Differentiation markers type I collagen, ALP, bone sialoprotein, osteocalcin, Runx2 and osterix were also decreased by Akt1 deficiency in osteoblasts (Fig 4B). This was a cell autonomous function of Akt1 since the impaired differentiation and matrix synthesis shown by ALP and Alizarin red stainings in the Akt1-/- osteoblast culture were restored by introduction of Akt1^CA^ (Fig 4C). Interestingly, the Akt1^CA^ introduction failed to commit immature mesenchymal cell lines C2C12 and C3H10T1/2 to osteoblastic lineage, although Runx2, a master transcription factor for osteoblastic differentiation, potently induced it (Supp. Fig S5).

These results suggest that Akt1 does not induce Runx2 expression but acts instead on differentiated osteoblasts that have the potency to express Runx2. Hence, as a possible mechanism underlying the osteogenic action of Akt1, we looked at its modulation of Runx2 function. Akt1 overexpression by Akt1^CA^ introduction into Runx2 stable transfectants of MC3T3-E1 cells enhanced the Runx2 activity on osteoblast differentiation and function, and vice versa, while Akt1 silencing by the siRNA introduction attenuated them (Fig 4D). Promoter activity of osteocalcin, a representative transcriptional target of Runx2, in Runx2-overexpressing MC3T3-E1 cells was also enhanced by Akt1^CA^ and suppressed by Akt1 siRNA (Fig 4E). Chromatin immunoprecipitation (ChIP) showed that the complex of Runx2 and osteocalcin promoter seen in WT osteoblasts disappeared in Akt1-/- osteoblasts (Fig 4F). Collectively, Akt1 is likely to enhance the Runx2-dependent osteoblast differentiation and function through enhancement of the DNA binding and the transcriptional activity.

### Akt1 deficiency impairs bone resorption via dysfunctions of osteoclasts and osteoblasts

**Figure 5:**
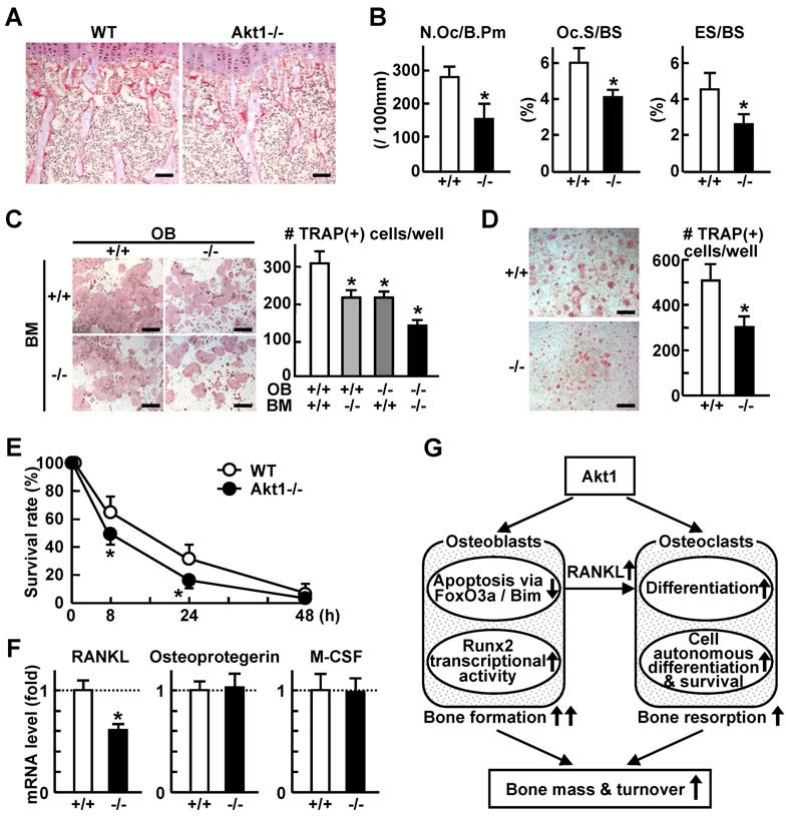
Akt1 deficiency impairs bone resorption via dysfunctions of osteoclasts and osteoblasts. (A) TRAP staining of proximal tibias of 8 week-old WT and Akt1-/- mice. Bars, 100 µm. (B) Bone resorption parameters in histomorphometric analysis at the secondary spongiosa of the proximal tibias. N.Oc/B. Pm, number of osteoclasts per 100 mm of bone perimeter; Oc.S/BS, osteoclast surface per bone surface; ES/BS, eroded surface per bone surface. Data are expressed as means (bars)±SEM (error bars) for 3–4 mice/group. *P<0.05 vs. WT (+/+). (C) The number of TRAP-positive multinucleated osteoclasts formed in the coculture of osteoblasts (OB) and bone marrow cells (BM) from WT or Akt1-/- littermates. Data are expressed as means (bars)±SEM (error bars) for 4 wells/group. *P<0.05 vs. WT. Bars, 500 µm. (D) The number of TRAP-positive multinucleated osteoclasts formed from BMM cultured in the presence of RANKL and M-CSF. Data are expressed as means (bars)±SEM (error bars) for 4 wells/group. *P<0.01 vs. WT. Bars, 200 µm. (E) Time course of survival rate of mature osteoclasts after deprivation of RANKL and M-CSF. Mature osteoclasts were formed from BMM in the presence of RANKL and M-CSF. Data are expressed as means (symbols)±SEM (error bars) for 4 wells/group. *P<0.05 vs. WT. (F) mRNA levels of RANKL, osteoprotegerin, and M-CSF in cultured osteoblasts by real-time RT-PCR. Data are normalized to those of β-actin and are expressed as means (bars)±SEM (error bars) of the relative amount compared to the WT culture. *P<0.01 vs. WT. (G) Schematic diagram of the mechanisms underlying the Akt1 function to maintain bone mass and turnover. Akt1 suppresses the susceptibility to mitochondria-dependent apoptosis of osteoblasts by inhibiting the FoxO3a nuclear entry and the Bim transactivation, and stimulates the differentiation and function by enhancing the Runx2 transcriptional activity, resulting in increased bone formation. Akt1 also induces RANKL expression in osteoblasts to support osteoclast differentiation, and shows cell-autonomous defects in osteoclasts to stimulate the differentiation and survival, resulting in increased bone resorption.

Finally, we investigated the role of Akt1 in bone resorption. The number of osteoclasts determined by tartrate-resistant acid phosphatase (TRAP) staining was reduced in the Akt1-/- bone in vivo (Fig 5A), which was confirmed by decreased bone resorption parameters in the histomorphometric analysis (Fig 5B). Osteoclastogenesis in the ex vivo co-culture was reduced when either osteoblasts or bone marrow cells were derived from Akt1-/- mice, and was additively reduced when both were from Akt1-/- (Fig 5C). This indicates that Akt1 is necessary for osteoclast precursors in a cell autonomous manner and for osteoblasts to support osteoclast differentiation. Osteoclastogenesis in the culture of osteoclast-progenitor BMM derived from Akt1-/- was suppressed even in the presence of soluble receptor activator of nuclear factor κB ligand (RANKL) and M-CSF (Fig 5D). Furthermore, the survival of mature osteoclasts formed from Akt1-/- BMM was also impaired (Fig 5E), confirming that the intrinsic Akt1 in osteoclastic cells is necessary to maintain bone resorptive function. Contrarily, decreased expression of RANKL, but not osteoprotegerin or M-CSF, in Akt1-/- osteoblasts (Fig 5F) explains the contribution of Akt1 in osteoblasts to osteoclastic bone resorption.

Taken together, Akt1 deficiency may cause impairment of bone resorption via the cell autonomous dysfunction in osteoclasts and the cell non-autonomous inhibition of osteoclastogenesis due to reduced RANKL expression in osteoblasts.

## Discussion

### Physiological roles of Akt1 in bone

The present study initially analyzed the bones of mice lacking Akt1 and found that the deficiency caused osteopenia with a low turnover state. Further in vivo and ex vivo analyses of osteoblasts and osteoclasts revealed that the Akt1 deficiency caused impairments of both bone formation and bone resorption via respective cell autonomous mechanisms (Fig 5G). The imbalance between formation and resorption, the underlying mechanism of which remains to be clarified, may cause osteopenia with a low bone turnover.

### Akt1 in osteoblasts

Akt1 deficiency in osteoblasts caused three cell autonomous abnormalities: increased susceptibility to apoptosis, suppressed differentiation and function, and decreased RANKL expression to support osteoclastogenesis. Most notably among them, the enhanced apoptosis in Akt1-/- osteoblasts was shown to be mitochondria-dependent via the FoxO3a nuclear entry and the Bim transactivation. A recent report on mice with osteoblast-specific deletion of phosphatase and tensin homolog (Pten) demonstrated reduced osteoblast apoptosis and increased bone mass by constitutive activation of Akt signaling, corresponding to the present finding, although the downstream molecular mechanism remained unclarified [18]. The candidates of substrates of Akt1 included caspase-9, Bad, and FoxOs whose involvement in mitochondria-dependent apoptosis had been established in other cells [9]. Although caspase-9 functions as one of the most important effectors in the apoptosis, the lack of a minimal substrate consensus sequence for Akt [9] led us to exclude this from the candidates. A proapoptotic molecule Bad was expressed in osteoblasts, but the phosphorylation could not be detected. FoxO proteins identified in mammals, FoxO1, FoxO3a and FoxO4, constitute a newly characterized subfamily of the Forkhead/winged helix group of transcription factors and play a predominant role in mediating various functions of the PI3K/Akt pathway [16], [17]. Although disruption of the genes in mice has revealed the necessities of FoxO1 in embryonic vessel formation and FoxO3a in ovarian follicular development [19], their roles in bone metabolism remained unknown. This study showed expressions of FoxO1 and FoxO3a, but not FoxO4, in osteoblasts, and functional involvement of FoxO3a in their survival as a phosphorylation target of Akt1. Contrarily, the nuclear entry of FoxO1 after serum deprivation was independent of Akt1 (Fig 2G), implicating that different isoforms of Akt may have specific preference to FoxO proteins as the substrates. In fact, similarly to Akt2, FoxO1 is expressed predominantly in insulin target tissues and regulates their insulin resistance [16].

Bim, Bcl-2-interacting mediator of cell death, was shown to be a transcriptional target of FoxO3a that lies downstream of the Akt1 anti-apoptotic signaling in osteoblasts. Proapoptotic activity of Bim is regulated both transcriptionally and post-transcriptionally [20], and the transcriptional induction is known to be mediated by FoxOs under the apoptotic stimulation in other cells [17], [21]–[23]. Studies on knockout mice have revealed that Bim is essential for apoptosis of lymphocytes, myeloid cells, neurons, and osteoclasts [24], [25]. Regarding osteoblast apoptosis, the involvement of Bcl-2 family proteins such as Bax and Bcl-2 has been reported [7], [26]. Because the changes in protein levels of these molecules are crucial for osteoblast apoptosis, these changes seem to be independent of the Bim regulation.

Suppressed differentiation and function in Akt1-/- osteoblasts was shown to be mediated at least partly by modulating the Runx2 activity. The present results using Akt1-null materials confirmed a previous report by Fujita et al. that gain- and loss-of-functions of PI3K/Akt signaling regulate the DNA binding and transactivity of Runx2 in cultures of osteoblastic cells [27]. Since Runx2 does not have a consensus sequence for Akt phosphorylation, Akt is not likely to phosphorylate Runx2 directly, but may regulate the activity or stability of co-activators or co-repressors of Runx2. However, the fact that Akt1-/- mice did not exhibit cleidocranial dysplasia (Supp. Fig S2B), the characteristic phenotype of Runx2+/− mice, indicates that Akt1 is not a crucial regulator of the Runx2-dependent bone formation in vivo.

### Akt1 in osteoclasts

Besides the defects in osteoblasts, the deficiency of Akt1 in osteoclasts caused impairment of bone resorption via the cell autonomous dysfunction. To date, the role of Akt signaling in osteoclasts has been controversial. RANKL and M-CSF have been reported to promote the osteoclast survival in part by activating the Akt pathway [28], [29]. Contrarily, Akt is shown to be dispensable for the survival by knockdown experiment using Akt1/Akt2 siRNA, but is necessary for the proliferation and differentiation [30]. Our results using ex vivo cultures of isolated Akt1-/- osteoclasts have provided genetic evidence that Akt1 signaling promotes the differentiation and survival. The regulation of differentiation might be dependent on the DNA binding of NFκB, since previous knockdown studies of Akt1 and/or Akt2 showed inhibition of RANKL-induced NFκB p50 DNA-binding activity via IκB kinase α [30].

By analogy with osteoblasts, the enhanced osteoclast survival by Akt1 might be mediated by the FoxO3a/Bim axis, since Bim is reported to be critical for osteoclast apoptosis [25]. However, this is dependent on post-transcriptional regulation by ubiquitylation and proteasomal degradation of Bim mediated by ERK pathway, but not on transcriptional regulation as seen in the case of osteoblast apoptosis. In addition, Bim in osteoclasts stimulates not only the apoptosis, but also the bone resorptive activity. If Bim is enhanced in Akt1-/- osteoclasts just as in the osteoblasts, the bone resorption parameters like eroded surface are assumed to increase. Hence, the mechanism by which Akt1 suppresses apoptosis in osteoclasts is likely to be different from that in osteoblasts. The cell autonomous mechanism of Akt1 in osteoclasts is the next task we intend to pursue.

### Akt1 as a mediator of bone anabolic signaling

Akt1 may mediate the osteoblastic bone formation by IGF-I and insulin, since their effects on osteoblast survival and differentiation were impaired in Akt1-/- osteoblasts (Fig 2C, 4A). Contrarily, although BMP-2 induced phosphorylation of the entire Akt (Supp. Fig S1A), the stimulated osteoblast differentiation was not affected by the Akt1 deficiency (Fig 4A), suggesting the mediation of other Akt isoforms or other pathways in the BMP-2 signaling. IGF-I is known to function as a potent bone anabolic factor via autocrine/paracrine and endocrine mechanisms [4], since skeletal and serum IGF-I levels were positively correlated with bone density between two inbred strains of mice [31]. Serum IGF-I levels of Akt1-/- mice were comparable to that of WT in the age from 8 to 16 weeks (data not shown), suggesting the absence of reduced IGF-I secretion or systemic compensation for impaired IGF-I signaling. Hence, the decreased bone formation in vivo in Akt1-/- mice may be at least in part due to the deficit of anabolic IGF-I signaling. Considering that Akt1; Akt2 double-knockout mice and IGF-I receptor knockout mice exhibit similar phenotypes [14], [32], Akt may be the main mediator of the IGF-I signaling. Bone anabolic action of other hormones like parathyroid hormone, growth hormone, and thyroid hormone are reported to be mediated by IGF-I signaling [33]–[36], and it is possible that the IGF-I/Akt1 pathway might be a common pathway for actions of these major hormones in bone.

As presented herein, Akt1, a multifaceted kinase which mediates various kinds of upstream signals to a diverse spectrum of substrates, plays multiple roles in bone cells as well as other cell types reported. Although it seems difficult to target this molecule directly in order to yield novel therapeutics for bone disorders because of its ubiquitous expression and diverse functions, further understanding of the molecular network related to Akt1 will greatly help us to unravel the complex mechanism modulating bone remodeling.

## Materials and Methods

### Mice

The generation of Akt1-/- mice was described previously [11]. All mice were maintained in the C57BL/6 background with a standard diet. In each experiment, homozygous WT and Akt1-/- mice that were littermates generated from the intercross between heterozygous mice were compared. All experiments were performed on 8-week-old male mice unless otherwise described, according to the protocol approved by the Animal Care and Use Committee of the University of Tokyo.

### Radiological analyses

Plain radiographs were taken using a soft X-ray apparatus (CMB-2, SOFTEX), and the BMD was measured by dual energy X-ray absorptiometry (DXA) using a bone mineral analyzer (PIXImus Densitometer, GE Medical Systems). CT scanning of the femurs was performed using a composite X-ray analyzer (NX-CP-C80H-IL, Nittetsu ELEX Co.), and reconstructed into a 3D feature by the volume-rending method (VIP-Station, Teijin System Technology). pQCT scan was performed at the metaphysis 1.4 mm above the distal growth plate and at the mid-shaft of femurs.

### Histological analyses

For toluidine blue staining, samples were fixed with 70% ethanol, embedded in methyl methacrylate, and sectioned in 6-µm slices. Histomorphometric analyses were performed as described [37] in the growth plate and secondary spongiosa (1.2 mm in length from 0.5 mm below the growth plate) of the proximal tibias, according to the ASBMR nomenclature report [38]. For double labeling of the mineralization front, mice were injected subcutaneously with 16 mg/kg body weight of calcein at 5 d and 1 d before sacrifice. TRAP-positive cells were stained at pH 5.0 in the presence of L (+)-tartaric acid using naphthol AS-MX phosphate (Sigma-Aldrich) in *N,N*-dimethyl formamide as the substrate. Apoptotic osteoblasts were detected by TUNEL method using an ApopTag Peroxidase In Situ Apoptosis Detection Kit (Chemicon) on paraffin-embedded sections of neonate mice. The percentage of apoptotic cells was calculated by dividing the number of TUNEL-positive cells by the number of counted cells. A total of at least 400 osteoblasts were counted in each section, and five to seven sections per group were analyzed.

### Osteoblastic cell cultures and assays

For osteoblast cultures, calvariae of neonatal mice were digested by 0.1% collagenase and 0.2% dispase 5 times, and cells isolated by the last 3 digestions were combined and cultured in α-minimal essential medium (α-MEM) containing 10% FBS. Mouse osteoblastic MC3T3-E1 cells were cultured in the same way. Immature mesenchymal C2C12 cells and C3H10T1/2 cells were cultured in Dulbecco's modified Eagle's medium (DMEM) supplemented with 10% FBS. Cell proliferation was determined using a BrdU Labeling and Detection Kit III (Roche Diagnostics). After 24 h culture in the presence and absence of insulin (100 nM), IGF-I (10 nM), or FGF-2 (1 nM), the cells were labeled with BrdU for an additional 4 h, and BrdU uptake was detected following the manufacturer's instructions. To determine the osteoblast apoptosis, the culture medium was changed to a serum-free one with and without insulin or IGF-I, and total RNA or protein was collected after designated times for subsequent assays. Cell survival was determined by counting viable cell numbers using the Cell Counting Kit-8 (Dojindo Molecular Technologies). Apoptotic osteoblasts were further detected by TUNEL staining as described above. Caspase-3 activity was measured via colorimetric detection of the cleavage of caspase-specific substrates using an APOPCYTO Caspase-3 Colorimetric Assay Kit (MBL International Co.). For ALP activity measurement, primary osteoblasts were inoculated at a density of 5×10^4^ cells/well in a 24-multiwell plate and cultured in α-MEM containing 10% FBS and 50 µg/ml ascorbic acid. After 14 d of culture in the presence and absence of insulin (100 nM), IGF-I (10 nM), or BMP-2 (30 ng/ml), cells were sonicated in 10 mM Tris-HCl buffer (pH 8.0) containing 1 mM MgCl_2_ and 0.5% Triton X-100. ALP activity in the lysate was measured using an ALP Kit (Wako Pure Chemical). Protein concentrations of cell lysates were measured with a Protein Assay Kit II (BIO-RAD). For ALP staining, cells were fixed in 70% ethanol and stained for 10 min with a solution containing 0.01% naphthol AS-MX phosphate, 1% *N,N*-dimethyl formamide, and 0.06% fast blue BB (Sigma-Aldrich). For Alizarin red S and von Kossa staining, osteoblasts were inoculated at a density of 1×10^5^ cells/well in a 12-multiwell plate in α-MEM containing 10% FBS and 50 µg/ml ascorbic acid and 10 mM β-glycerophosphate (Sigma-Aldrich). On day 21 after confluency, cultured cells were fixed in 10% buffered formalin and stained for 10 min with 2% Alizarin red S (pH 4.0) (Sigma-Aldrich). For von Kossa staining, cells were fixed with 100% ethanol at room temperature for 15 min, stained with 5% silver nitrate solution under ultraviolet light for 10 min, and incubated for 5 min with 5% sodium thiosulfate solution.

### Assays for osteoclastic cells

To study the role of Akt1 intrinsic to osteoclastic cells, we used a coculture of osteoblasts and bone marrow cells and the M-CSF-dependent BMM culture system as described previously [39]. Bone marrow cells were collected from long bones of 8-wk-old WT or Akt1-/- littermates. TRAP-positive multinucleated osteoclasts were generated by coculturing osteoblasts (1×10^4^ cells/well) and bone marrow cells (5×10^5^ cells/well) derived from either WT or Akt1-/- littermates in a 24-multiwell plate in α-MEM containing 10% FBS, 1,25(OH)_2_D_3_ (10 nM), and prostaglandin E_2_ (100 nM). After 6 d, cells positively stained for TRAP containing more than three nuclei were counted as osteoclasts. In the M-CSF-dependent BMM culture system, bone marrow cells from WT or Akt1-/- mice were seeded at a density of 3×10^5^ cells/well in a 24-multiwell plate and cultured in α-MEM containing 10% FBS with M-CSF (R&D Systems, 100 ng/ml) for 3 d. To generate mature osteoclasts, adherent cells (BMM) were further cultured with M-CSF (10 ng/ml) and soluble RANKL (Wako Pure Chemical, 100 ng/ml) for 3 additional days, then the number of TRAP-positive osteoclasts was counted. To determine the survival, the mature osteoclasts were deprived of M-CSF/soluble RANKL and cultured for an additional 48 h. After 8, 24, and 48 h, the number of TRAP-positive and trypan blue-negative osteoclasts was counted.

### Real-time quantitative RT-PCR

Total RNA was extracted with ISOGEN (Wako Pure Chemical), and an aliquot (1 µg) was reverse-transcribed using a PrimeScript RT reagent Kit (Takara Bio) to make single-stranded cDNA. PCR was performed on an ABI Prism 7000 Sequence Detection System (Applied Biosystems) using QuantiTect SYBR Green PCR Master Mix (QIAGEN) according to the manufacturer's instructions. All reactions were run in triplicate. After data collection, the mRNA copy number of a specific gene in total RNA was calculated with a standard curve generated with serially diluted plasmids containing PCR amplicon sequences, and normalized to the rodent total RNA (Applied Biosystems) with mouse β-actin as an internal control. Standard plasmids were synthesized with a TOPO TA Cloning Kit (Invitrogen), according to the manufacturer's instruction. Primer sequences are stated in the Supporting Protocol S1 online.

### Western blot analyses

Cells were washed twice with ice-cold PBS, and proteins were extracted with M-PER (Pierce Chemical) or NE-PER (Pierce Chemical), according to the manufacturer's instructions. To detect the phosphorylation of FoxO3a, the lysates were treated with lambda protein phosphatase (New England Bio Labs). For Western blot analysis, lysates were fractionated by SDS-PAGE with 7.5–15% Tris-Glycin gradient gel or 15% Tris-Glycin gel, and transferred onto nitrocellulose membranes (BIO-RAD). After blocking with 6% milk/TBS-T, membranes were incubated with primary antibodies to pan-Akt, phospho-Akt, Akt1, Akt2, FoxO1, cleaved caspase-3, Bax, Bcl-2 (Cell Signaling Technology), FoxO3a (Upstate), Bim (BD Parmingen), Bcl-x_L_ (Santa Cruz Biotechnology), and β-actin (Sigma-Aldrich), followed with HRP-conjugated goat anti-mouse IgG and goat anti-rabbit IgG (Promega). Immunoreactive bands were visualized with ECL Plus (Amersham), according to the manufacturer's instructions.

### Luciferase assays

MC3T3-E1 cells were plated onto 24-well plates, then subsequently transfected in triplicate with 0.1 µg of the reporter plasmid, 0.2 µg of effector plasmids, and 8 ng of pRL-TK vector (Promega) for internal control, by using FuGENE6 (Roche Diagnostics). The amount of total DNA in each well was adjusted to be equal. Details are described in Supporting Protocol S1 online. The luciferase assay was performed 48 h after transfection with a dual-luciferase reporter assay system and GloMax™ 96 Microplate Luminometer (Promega). Firefly output was normalized to Renilla output to control for transfection efficiency.

### ChIP assay

ChIP assay was performed with a ChIP Assay Kit (Upstate), according to the manufacturer's instructions. PCR was performed to amplify the promoter region (−471/−67) of osteocalcin gene containing the OSE2 site which Runx2 is reported to bind [40]. Primer sequences are given in Supporting Protocol S1 online.

### Statistical analyses

Means of groups were compared by ANOVA and significance of differences was determined by post-hoc testing with Bonferroni's method.

## Supporting Information

Figure S1 Phosphorylation and expression pattern of Akt isoforms in bone cells. (A) Time course of phosphorylated total Akt (P-Akt) and total Akt (Akt) levels determined by Western blotting in cultured mouse calvarial osteoblasts (OB) after stimulation with IGF-I or BMP-2, and in cultured bone marrow macrophages (BMM) and mature osteoclasts (OC) after stimulation with M-CSF. (B) Expressions of each Akt isoform in the cells above determined by quantitative real-time RT-PCR analysis using the same amount of template cDNA. Data are expressed as means (bars)±SEM (error bars) of the relative amount of mRNA as compared to that of Akt1.
(0.49 MB TIF)

Figure S2 Akt1-/- mice showed growth retardation. (A) Body weight and naso-anal length of WT (+/+) and Akt1-/- littermates. Data are expressed as means (symbols)±SEM (error bars) for 4–10 mice/group. *P<0.05 vs. WT. (B) Plain X-ray images of the whole body of representative male littermates at 8 weeks of age. Bar, 1 cm.
(1.47 MB TIF)

Figure S3 FoxO3a translocated into nucleus after serum deprivation. Time course of subcellular localization of GFP-tagged FoxO3a after serum deprivation in MC3T3-E1 cells. Bar, 20 µm.
(2.16 MB TIF)

Figure S4 Effects of Actinomycin D, insulin, and IGF-I on Bim expression after serum deprivation. (A) Time course of Bim mRNA level determined by real-time RT-PCR after serum deprivation in cultured calvarial osteoblasts with and without Actinomycin D (1 µM). Data are normalized to those of β-actin and are expressed as means (symbols)±SEM (error bars) of the relative amount compared to time 0. (B) Time course of Bim protein level determined by Western blotting after serum deprivation in cultured calvarial osteoblasts with and without Actinomycin D (1 µM). (C) Time course of Bim mRNA level determined by real-time RT-PCR after serum deprivation in cultured calvarial osteoblasts with and without insulin (100 nM), IGF-I (10 nM), or FBS (10%). Data are normalized to those of β-actin and are expressed as means (symbols)±SEM (error bars) of the relative amount compared to time 0.
(0.38 MB TIF)

Figure S5 Akt1 did not affect osteoblastic differentiation of immature mesenchymal cell lines. ALP staining of cultured C2C12 cells and C3H10T1/2 cells that were adenovirally transfected with GFP, Akt1^CA^, or Runx2.
(1.52 MB TIF)

Protocol S1 (0.10 MB DOC)
