# Supplementary figures and images for: Akt1 in Osteoblasts and Osteoclasts Controls Bone Remodeling
Source: PLoS One. 2007 Oct 24;2(10):e1058. doi: 10.1371/journal.pone.0001058 (PMC2020440; doi:10.1371/journal.pone.0001058)

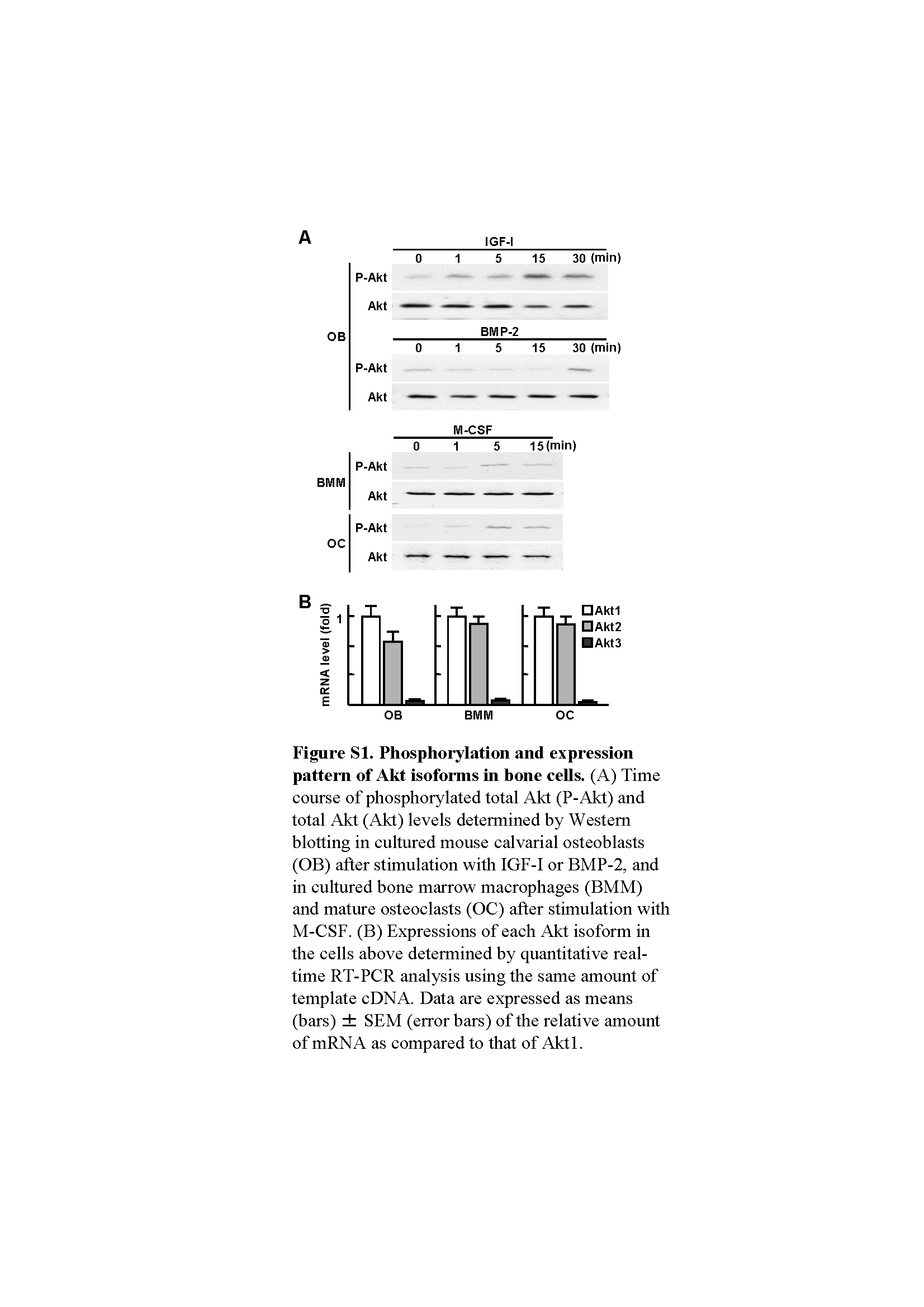

Supplement: Figure S1 — Phosphorylation and expression pattern of Akt isoforms in bone cells. (A) Time course of phosphorylated total Akt (P-Akt) and total Akt (Akt) levels determined by Western blotting in cultured mouse calvarial osteoblasts (OB) after stimulation with IGF-I or BMP-2, and in cultured bone marrow macrophages (BMM) and mature osteoclasts (OC) after stimulation with M-CSF. (B) Expressions of each Akt isoform in the cells above determined by quantitative real-time RT-PCR analysis using the same amount of template cDNA. Data are expressed as means (bars)±SEM (error bars) of the relative amount of mRNA as compared to that of Akt1. (0.49 MB TIF) [file pone.0001058.s001.tif]

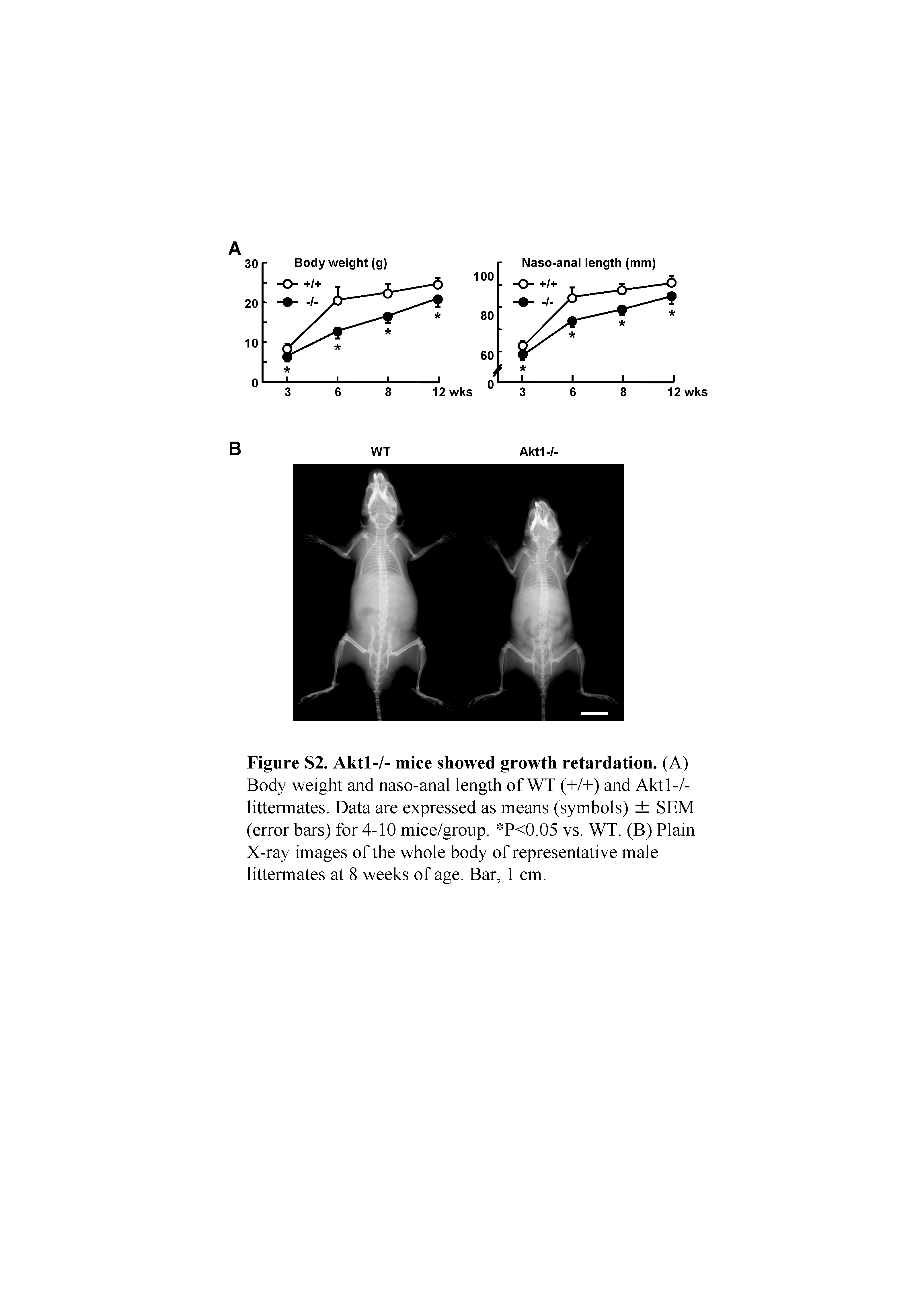

Supplement: Figure S2 — Akt1-/- mice showed growth retardation. (A) Body weight and naso-anal length of WT (+/+) and Akt1-/- littermates. Data are expressed as means (symbols)±SEM (error bars) for 4–10 mice/group. *P<0.05 vs. WT. (B) Plain X-ray images of the whole body of representative male littermates at 8 weeks of age. Bar, 1 cm. (1.47 MB TIF) [file pone.0001058.s002.tif]

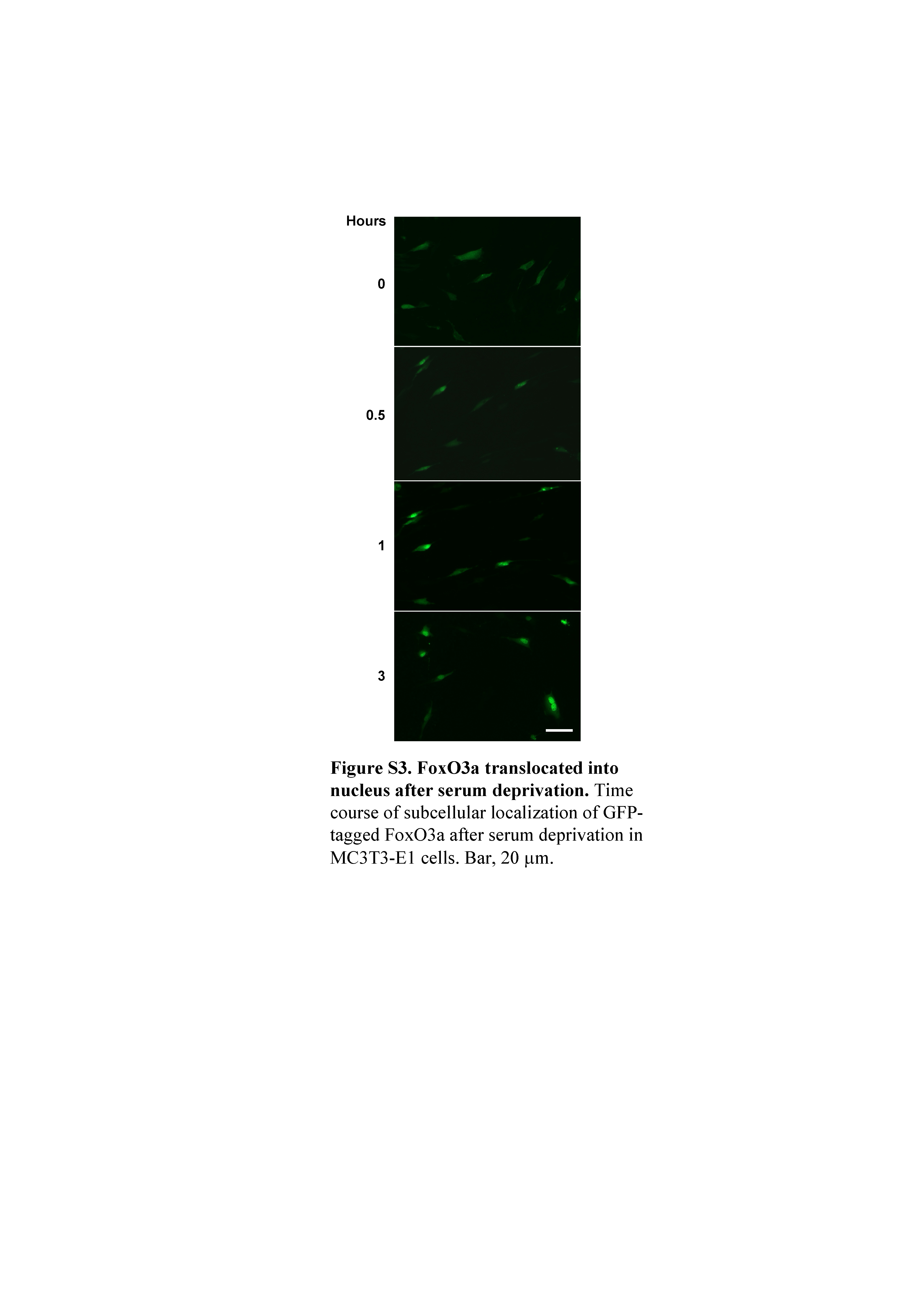

Supplement: Figure S3 — FoxO3a translocated into nucleus after serum deprivation. Time course of subcellular localization of GFP-tagged FoxO3a after serum deprivation in MC3T3-E1 cells. Bar, 20 µm. (2.16 MB TIF) [file pone.0001058.s003.tif]

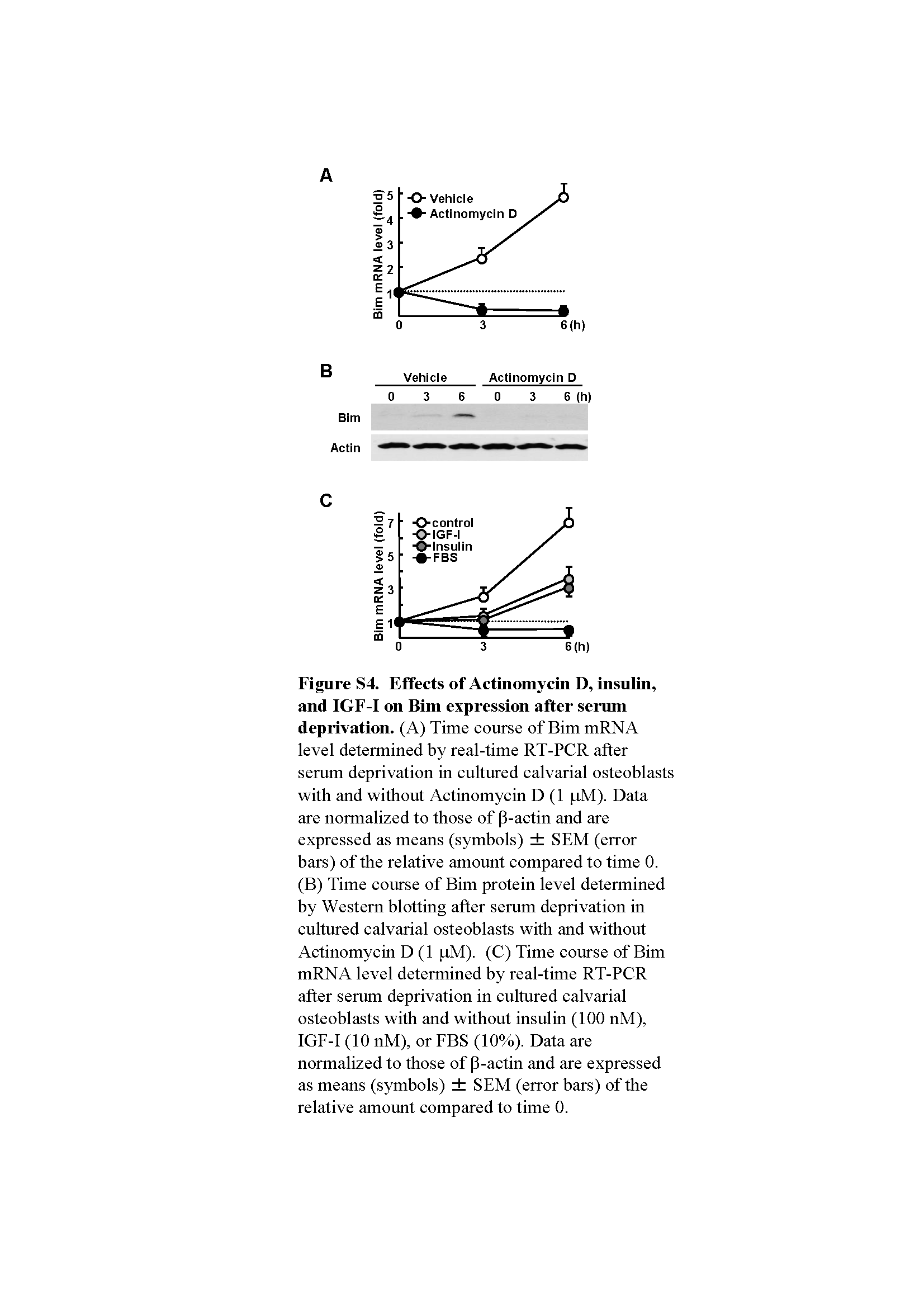

Supplement: Figure S4 — Effects of Actinomycin D, insulin, and IGF-I on Bim expression after serum deprivation. (A) Time course of Bim mRNA level determined by real-time RT-PCR after serum deprivation in cultured calvarial osteoblasts with and without Actinomycin D (1 µM). Data are normalized to those of β-actin and are expressed as means (symbols)±SEM (error bars) of the relative amount compared to time 0. (B) Time course of Bim protein level determined by Western blotting after serum deprivation in cultured calvarial osteoblasts with and without Actinomycin D (1 µM). (C) Time course of Bim mRNA level determined by real-time RT-PCR after serum deprivation in cultured calvarial osteoblasts with and without insulin (100 nM), IGF-I (10 nM), or FBS (10%). Data are normalized to those of β-actin and are expressed as means (symbols)±SEM (error bars) of the relative amount compared to time 0. (0.38 MB TIF) [file pone.0001058.s004.tif]

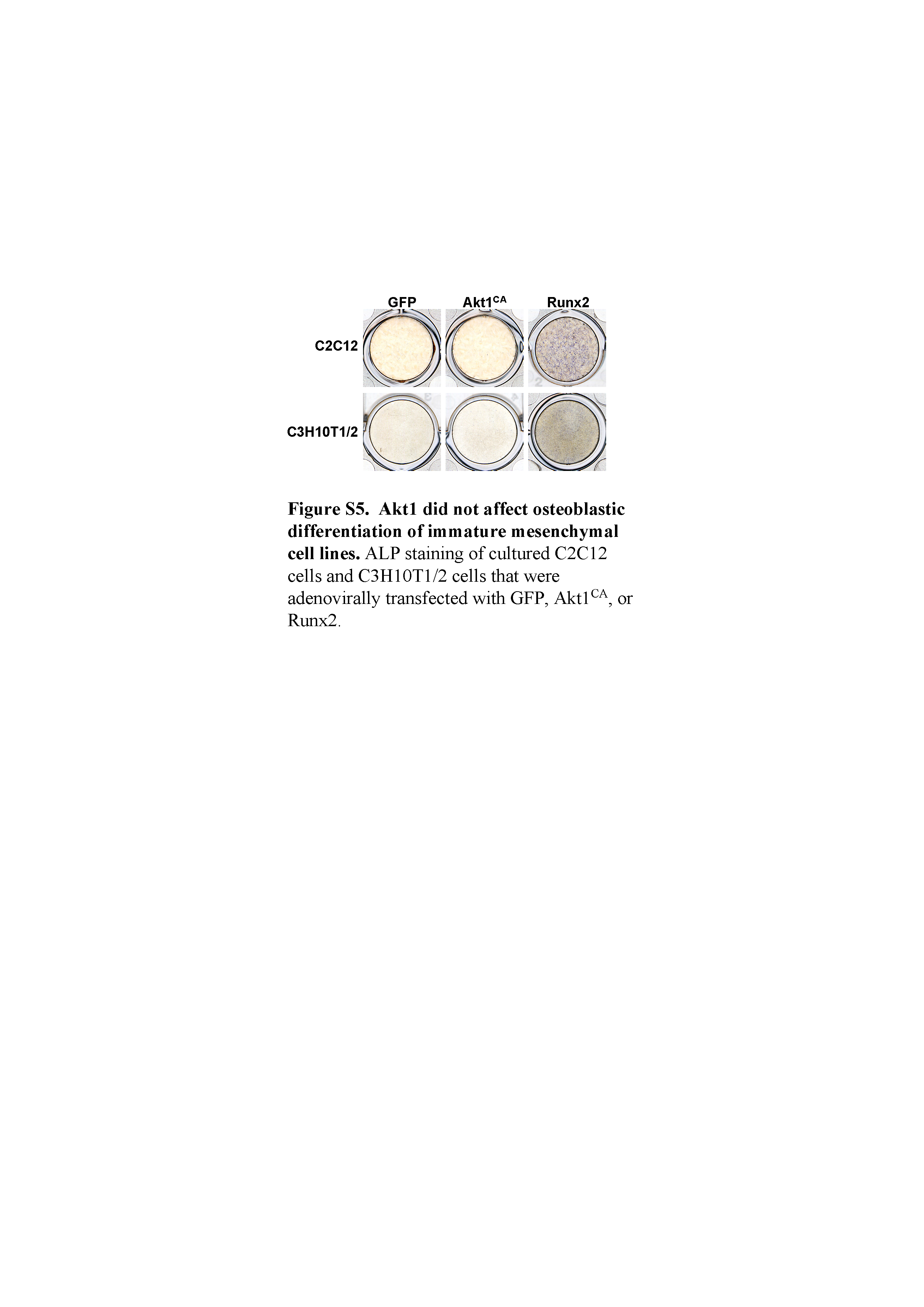

Supplement: Figure S5 — Akt1 did not affect osteoblastic differentiation of immature mesenchymal cell lines. ALP staining of cultured C2C12 cells and C3H10T1/2 cells that were adenovirally transfected with GFP, Akt1CA, or Runx2. (1.52 MB TIF) [file pone.0001058.s005.tif]
