# Supplementary material for: Akt1 in Osteoblasts and Osteoclasts Controls Bone Remodeling
Source: PLoS One. 2007 Oct 24;2(10):e1058. doi: 10.1371/journal.pone.0001058 (PMC2020440; doi:10.1371/journal.pone.0001058)
Supplement: Protocol S1 — (0.10 MB DOC) [file pone.0001058.s006.doc]

**SUPPORTING PROTOCOL**

**Construction of expression vectors and viruses**. Full-length Akt1, FoxO1, FoxO3a, FoxO4, Runx2, and Cbfb cDNA were amplified by PCR and cloned into pCMV-HA vector (Clontech). Expression vector for the constitutively active form of Akt1 (Akt1^CA^) was produced by PCR to insert the myristylation sequence in N-terminus of HA-Akt1 as previously reported [1,2,3]. Expression vector for a dominant negative form of FoxO3a (FoxO3a^DN^) was produced by deleting N-terminus and C-terminus, which flanked DNA binding domain of FoxO3a, from pCMV-HA-FoxO3a as previously reported [4].

For luciferase assay, the 2-Kb fragment containing the region 5’ to the Bim initiator codon was obtained by PCR using human genomic DNA as a template and was subcloned into pGL3-Basic vector (Promega). The luciferase reporter constructs containing a 1,050 bp of rat osteocalcin 5’-end flanking region was a generous gift from Dr. H. Takayanagi at Tokyo Medical & Dental University. Osteocalcin promoter activity was analyzed in retroviral Runx2 transfectants of MC3T3-E1 cells.

For production of retrovirus, cDNA of FoxO3a, FoxO3a^DN^, Akt1^CA^, or Runx2 was inserted into pMx vector [5]. Production of retrovirus vectors was performed as previously reported [6]. Briefly, plat-E cells (2 × 10^6^ cells) were plated in 60-mm dishes, and transfected with 2 μg pMx vector using Fugene (Roche) on the following day. After 24 h, the medium was replaced with fresh medium, which was collected and used as the retrovirus supernatant 48 h after the transfection. Puromycine resistance gene and blasticidin resistance gene were inserted into pMx vector for selection of stable cells.

For gene silencing, RNAi sequence was designed for the genes of mouse Akt1, mouse Bim, and GFP. Targeting sequences are GCACCTTTATTGGCTACAAGG for siAkt1 and GGAGACGAGTTCAACGAAACT, GGCCAAGCAACCTTCTGATGT, GCTGAAGACCACCCTCAAATG for siBim, GCTACGTCCAGGAGCGCACCA for siGFP. siRNA expression vectors for these genes were constructed with piGENEmU6 vector (iGENE Therapeutics) as previously reported [7]. For retrovirus expressing siRNA, U6 promoter and inserts in piGENE vectors were cloned into pMx vectors.

The adenovirus expressing GFP and Bcl-x_L_ was created as previously described [8]. Adenovirus expressing constitutively active mouse Akt1 (Akt1^CA^) was a generous gift from Dr. H. Katagiri at the University of Tokyo [2,3]. Adenovirus expressing mouse Runx2 was a generous gift from Dr. R. Nishimura at Osaka University, Japan. Adenoviruses were amplified in HEK293 cells and purified with AdenoX Virus Purification Kit (Clontech). Viral titers were determined by the end-point dilution assay. Adenovirus was used at 50 multiplicity of infection (MOI). Total MOI in each well was adjusted to be equal with the adenovirus encoding GFP gene.

**Primer information.** Each primer sequence of mouse target genes is described below.

5’-CCCTTCTACAACCAGGACCA-3’ and 5’-ATACACATCCTGCCACACGA-3’ for Akt1 ;

5’-TCACTGACTTTGGCTTGTGC-3’ and 5’-TTGGGTCCTTCTTCAGCAGT-3’ for Akt2 ;

5’-CAGACACCCGATATTTTGATGA-3’ and 5’-CAGGCAAAGTCTAAGATGACAGTG-3’ for Akt3 ; 5’-CGTGGGAGTCTCAAAGGTGT-3’ and 5’-CGTTGTTGGTTTGAATGTGG-3’ for FoxO3a ; 5’-TTCGGAATGACCTCATGGAT-3’ and 5’-GCATCTTTGGACTGCTCCTC-3’ for FoxO1 ; 5’-CTCCCATCCCTAAGGTCCTG-3’ and 5’-AGTCCCTCACCATCCATGAG-3’ for FoxO4 ; 5’-GCCCAGACATTTGGTCAGTT-3’ and 5’-TGCACACACACACAGAGGAA-3’ for Bim ; 5’-CCCCAGTACACCCTCTGAAA-3’ and 5’-GCTGGTTGTTGCAAGA-3’ for Fas ligand ; 5’-GCTGGGACACTTTTGTGGAT-3’ and 5’-TGTCTGGTCACTTCCGACTG-3’ for Bcl-x_L_ ;

5’-ACGTCCTGGTGAAGTTGGTC-3’ and 5’-CAGGGAAGCCTCTTTCTCCT-3’ for α1 type I collagen (COL1A1); 5’-GCTGATCATTCCCACGTTTT-3’ and

5’-CTGGGCCTGGTAGTTGTTGT-3’ for ALP; 5’-CAGAGGAGGCAAGCGTCACT-3’ and 5’-CTGTCTGGGTGCCAACACTG-3’ for bone sialoprotein (BSP) ; 5’-AAGCAGGAGGGCAATAAGGT-3’ and 5’-TTTGTAGGCGGTCTTCAAGC-3’ for Osteocalcin; 5’-CCCAGCCACCTTTACCTACA-3’ and 5’-TATGGAGTGCTGCTGGTCTG-3’ for Runx2; 5-TGAGGAAGAAGCCCATTCAC-3’ and 5’-ACTTCTTCTCCCGGGTGTG-3’ for Osterix; 5’-AGATGTGGATCAGCAAGCAG-3’ and 5’-GCGCAAGTTAGGTTTTGTCA-3’ for β-actin ; 5’-CCTGAGGCCCCAGCCATTT-3’ and 5’-CTTGGCCCAGCCTCGAT-3’ for RANKL; 5’-CTGCTGAAGCTGTGGAAACA-3’ and 5’-GCTCGATTTGCAGGTCTTTC-3’ for Osteoprotegerin ; 5’-CTGGAAGGAGGATCAGCAAG-3’ and 5’-ATGTCTGAGGGTTTCGATGG-3’ for M-CSF.

The primer sequences for PCR to amplify the promoter region (-471/-67) of Osteocalcin gene in ChIP assay were 5’-CTGAACTGGGCAAATGAGGACA-3’ and 5’-AGGGGATGCTGCCAGGACTAAT-3’.

**References**

1. Resh MD (1994) Myristylation and palmitylation of Src family members: the fats of the matter. Cell 76: 411-413.

2. Katagiri H, Asano T, Ishihara H, Inukai K, Shibasaki Y, et al. (1996) Overexpression of catalytic subunit p110alpha of phosphatidylinositol 3-kinase increases glucose transport activity with translocation of glucose transporters in 3T3-L1 adipocytes. J Biol Chem 271: 16987-16990.

3. Katagiri H, Asano T, Inukai K, Ogihara T, Ishihara H, et al. (1997) Roles of PI 3-kinase and Ras on insulin-stimulated glucose transport in 3T3-L1 adipocytes. Am J Physiol 272: E326-331.

4. Gilley J, Coffer PJ, Ham J (2003) FOXO transcription factors directly activate bim gene expression and promote apoptosis in sympathetic neurons. J Cell Biol 162: 613-622.

5. Kitamura T (1998) New experimental approaches in retrovirus-mediated expression screening. Int J Hematol 67: 351-359.

6. Morita S, Kojima T, Kitamura T (2000) Plat-E: an efficient and stable system for transient packaging of retroviruses. Gene Ther 7: 1063-1066.

7. Miyagishi M, Taira K (2004) RNAi expression vectors in mammalian cells. Methods Mol Biol 252: 483-491.

8. Ikeda T, Kamekura S, Mabuchi A, Kou I, Seki S, et al. (2004) The combination of SOX5, SOX6, and SOX9 (the SOX trio) provides signals sufficient for induction of permanent cartilage. Arthritis Rheum 50: 3561-3573.
